# System of Diseases of the Eye

**Published:** 1898-03

**Authors:** 


**System of Diseases of the Eye.** Edited by WILLIAM F. NORRIS, M.D., and CHARLES A. OLIVER, M.D. Vol. I.—*Embryology, Anatomy, and Physiology of the Eye.* Pp. xvii., 7–670. Philadelphia: J. B. Lippincott Company. 1897.

Notwithstanding the large amount of literature in the English language dealing with ophthalmology, there has hitherto been no complete work presenting anything like a full summary of our knowledge of the subject. It is a notorious fact that though an immense amount of useful work has been done during the last few years, yet there has been no very pronounced departure from old and accepted methods, and no very startling development in the science of ophthalmology. It has, so to speak, settled down to certain accepted facts and principles. The time is therefore particularly appropriate for the production of this "System of Diseases of the Eye," a comprehensive work which is to occupy four volumes.

The first volume, which is now under our consideration, contains eleven articles; of which two are by English authors, two by German writers, and the remainder by Americans who are well-known masters of the subjects they have undertaken. In the first article Dr. John A. Ryder, of Philadelphia, strikes what we take to be the key-note of the whole "System," and deals *ab initio* with every detail in the development, not only of the eyeball and its appendages, but also of the orbital nerves and optic tracts. He relates evidence confirming the view that the vertebrate's eye was functional as such long before it developed an optic stalk, or even was pushed out from the brain-wall. The paired eyes of the higher vertebrates were primarily nearly median in their position. The theory that the retina really represents a portion of the primitive brain-wall or cortex precociously projected outward upon the optic stalk is well traced; as also is that of Müller and others, that the optic *stalk* does not share in the formation of the nervous part of the eye, but is broken down in course of development, and only serves as a guide to the optic nerve-fibres as they grow backwards from the retina to the brain. Drawings from the recent work of Miss Platt on the origin of the orbital muscles are introduced, showing the manner in which "these outgrowths from the head-cavities grope their way through the mesoderm and embrace the eyeball, seeking, so to speak, for their proper points of insertion upon it."

The four chapters on the Anatomy and Histology of the Eye occupy more than half the book, and it would be impossible here to do justice to the numerous good points contained in them. The information is, of course, up to date and accurate, and moreover is clearly put. For instance, what many regard as a vague structure, the capsule of Tenon, is accurately and, by means of a simple diagram, intelligibly described. In the same way the canal of Schlemm, and its communications with the venous system on the one hand, and with the anterior chamber on the other, are admirably presented. A full account is given of Leber's experiments, showing that the spaces of Fontana cannot in the normal eye be injected from the anterior chamber with non-diffusible colouring matter such as Berlin blue, and proving that no "open communication" exists between the aqueous humour and the venous circulation in the canal of Schlemm. Further evidence is given which tends to prove that there is no absorption of the aqueous by the anterior surface of the iris. Amid so much minute detail the practical bearing and surgical importance of various points are not lost sight of, nor are matters of historical interest neglected. The ciliary muscle is traced from its discovery by Eustachius down to the latest details. The histological evidence of Juler, and the physiological experiments of Langley and Anderson, are related in support of the theory that dilatation of the pupil is due to radial muscular fibres, and this view must now be considered as definitely proved. In about fifty pages giving a very clear account of the structure of the retina we do not notice any important departure from already accepted teaching, and some of the illustrations have been familiar for many years. The theory of "twin cones" —the apex of one cone bent over in a loop to join the apex of a neighbouring rod (or, more rarely, cone)—is, of course, given; but the author does not express his opinion as to the accuracy of the investigations on which this view is based. In fact, we may say throughout this portion of the work, amid all the wealth of theory and research submitted to the reader, he is too frequently left to form his own opinion as to which view is the most tenable. We should prefer the writer to come forward not merely as an author but as an authority. The chapter by Dr. Alexander Hill on the anatomy of the intracranial portion of the visual apparatus does not suffer from this defect, and is exceedingly good and ably written. He assesses the value of experimental evidence with great clearness and judgment; but the diagrams in this chapter are meagre and scarcely up to the standard of the illustrations of the rest of the work. We are also surprised to find no mention of the cortical destination of the fibres of the macular bundle.

The section devoted to Congenital Malformations of the Eye deals with every known variety of abnormality in systematic order. The now generally accepted explanation of buphthalmos is well set forth, and the common error of regarding coloboma of the iris as due to persistence of the fœtal fissure is exposed and the correct explanation given.

The chapters on dioptrics, light perception, and binocular vision call for no special comment. They show that the present clinical tests for vision, complete as they are compared with those of twenty years ago, still leave much to be desired. Dr. William Thomson discusses at some length the various theories of colour vision, and inclines to the acceptance of the Young-Helmholtz theory. He adopts the view recently advocated by Professor Rutherford that the occasional limitation of colour blindness to one eye proves that the defect is retinal and not cortical. Seeing that it has been shown that there are special nerve endings for appreciating heat, cold, pain, and touch, special nerve endings for appreciating the various primary colours seem not improbable. The last thirty-three pages are devoted to the photo-chemistry of the retina. The characteristics of the various forms of retinal pigment are described, and interesting details given of recent research into the question of migration of the pigment and the shifting of the cones in the frog's retina under the influence of light. The variations in the electrical conditions of the optic nerve when the retina is subjected to light are fully discussed. But after reading this able article we must confess that we are nearly as much in the dark as ever as to how light-waves become converted into light-sensations.

It is inevitable that in a series of articles by various writers there should be much repetition, and we cannot help regretting that each article is not prefaced with a summary of its contents; the index, full, admirable, and accurate as it is, hardly compensates for the want of this. The printing and general get-up of the volume leave nothing to be desired, and the profusion of plates and diagrams renders it very complete. The editors and publishers have succeeded in a very ambitious undertaking, and deserve congratulation for producing a work which will be welcomed by every oculist conversant with the English language.

